# The Dynamic Codon Biaser: calculating prokaryotic codon usage biases

**DOI:** 10.1099/mgen.0.000663

**Published:** 2021-10-26

**Authors:** Brian Dehlinger, Jared Jurss, Karson Lychuk, Catherine Putonti

**Affiliations:** ^1^​ Bioinformatics Program, Loyola University Chicago, Chicago, IL 60660, USA; ^2^​ Department of Biology, Loyola University Chicago, Chicago, IL 60660, USA; ^3^​ Department of Computer Science, Loyola University Chicago, Chicago, IL 60660, USA; ^4^​ Department of Microbiology and Immunology, Loyola University Chicago, Stritch School of Medicine, Maywood, IL 60153, USA

**Keywords:** codon usage, codon bias, Dynamic Codon Biaser, prokaryotes

## Abstract

Bacterial genomes often reflect a bias in the usage of codons. These biases are often most notable within highly expressed genes. While deviations in codon usage can be attributed to selection or mutational biases, they can also be functional, for example controlling gene expression or guiding protein structure. Several different metrics have been developed to identify biases in codon usage. Previously we released a database, CBDB: The Codon Bias Database, in which users could retrieve precalculated codon bias data for bacterial RefSeq genomes. With the increase of bacterial genome sequence data since its release a new tool was needed. Here we present the Dynamic Codon Biaser (DCB) tool, a web application that dynamically calculates the codon usage bias statistics of prokaryotic genomes. DCB bases these calculations on 40 different highly expressed genes (HEGs) that are highly conserved across different prokaryotic species. A user can either specify an NCBI accession number or upload their own sequence. DCB returns both the bias statistics and the genome’s HEG sequences. These calculations have several downstream applications, such as evolutionary studies and phage–host predictions. The source code is freely available, and the website is hosted at www.cbdb.info.

## Data Summary

The DCB webserver is available at www.cbdb.info and source code is available at https://github.com/BrianDehlinger/DCB-Dynamic-Codon-Biaser.

**Impact Statement**

Codon usage bias is a key feature of many genomes. Within bacterial genomes, codon usage can emerge as a result of mutational bias and translational selection and has been associated with gene expression levels. Highly expressed genes often represent the strength of the bias within a given genome. We have developed a web tool – Dynamic Codon Biaser (DCB) – for users to calculate codon usage bias for any publicly available genome sequence (complete or draft) or upload their own sequence. Currently no tool exists for users to analyse individual strains or unpublished sequences. Thus, researchers can consider this important metric when analysing a bacterial genome. The source code for this tool is also publicly available.

## Introduction

In many bacterial genomes there is a preferential usage of certain codons over other synonymous codons for the same amino acid. Processes such as mutational bias and translational selection often cause these biases (see reviews [1, 2]). Bacteria have been shown to have varying degrees of codon usage bias, suggesting that there are varying amounts of translational selection among different bacteria. In fact, genes that undergo more translational selection often have a greater codon usage bias [3, 4], and codon usage has been optimized by prokaryotic species over time to improve their translation [5]. Further bolstering this theory, evidence shows that high codon usage bias correlates with high gene expression [6]. Transcript structure and codon usage have been found to have significant effects on both protein production, mRNA abundance and stability, and also bacterial growth rate [7–14]. Biases in di-codon usage also have been observed [15, 16] and usage differs between the coding sequences of highly and lowly abundant proteins [17]. Furthermore, codon usage optimization is well documented within bacteriophage species: phages frequently reflect the codon usage of their bacterial host [18–22]. While similarities in subsequence usage, including codon bias, have been used to predict a phage’s host species [23, 24], alone it has limited success [24]. This prompted our prior development of CBDB: the Codon Bias Database [25], which contained precomputed calculations of codon bias usage within the highly expressed genes (HEGs) of bacterial Reference sequence (RefSeq) genomes [26].

A number of different metrics have been proposed to quantify codon bias, including relative synonymous codon usage (RSCU) [27], the codon adaptation index (CAI) [3], the self-consistent codon index (SSCI) [28] and relative codon adaptation index (rCAI) [29]. Rather than looking at the codons themselves to ascertain biases, a second approach exists in which biases are assessed relative to individual tRNA abundances, the tRNA adaptation index (tAI) [30]. Several resources for examining codon usage bias already exist (Table 1), including five web resources: CoCoPUTs [31], CAIcal [32], HEG-DB [33], SMS and COUSIN [34]. These web tools can be categorized as either a database (CoCoPUTs and HEG-DB) or interactive analysis for user-supplied sequences (CAIcal, SMS and COUSIN). The CoCoPUTs database provides a graphical user interface (GUI) that allows a user to specify a taxonomic id or scientific name and returns codon usage tables, the effective number of codons (ENC), and codon pair usage calculated from NCBI complete genome sequences [35]. HEG-DB gives the CAI values for HEGs of 200 bacterial genomes [32, 33]. Our previous database, CBDB, included codon usage metrics for HEGs for hundreds of bacterial RefSeq genomes [25], although with the introduction of our new tool presented here, it is no longer available. While the other tools listed in Table 1 are also capable of calculating codon metrics for HEGs, they require users to identify HEG sequences and supply these gene sequences (and/or codon usage tables).

**Table 1.** Available tools for calculating codon usage metrics

Tool	Functionality	Availability	URL (citation)
CAIcal	Calculates CAI for provided gene sequences and codon usage tables	Web	http://genomes.urv.es/CAIcal/ [32]
CoCoPUTs	Database of codon-pair and dinucleotide statistics for all genomes in GenBank	Web	https://hive.biochemistry.gwu.edu/review/codon2 [31]
CodonW	Calculates codon metrics for user-selected gene set and correspondence analysis	Local installation	https://sourceforge.net/projects/codonw/
coRdon	Calculates codon bias statistics	R package	https://www.bioconductor.org/packages/devel/bioc/vignettes/coRdon/inst/doc/coRdon.html
COUSIN	Calculates codon usage for user-supplied sequences	Web or install	http://cousin.ird.fr/index.php [34]
EncPrime	Calculates ENC metric	Local installation	https://github.com/jnovembre/ENCprime
GCUA	Calculates codon metrics for user-selected gene set and correspondence analysis	Local installation	http://mcinerneylab.com/software/gcua/
HEG-DB	Database of CAI index of HEGs for 200 genomes	Web	http://genomes.urv.cat/HEG-DB/ [33]
SMS	Calculates codon metrics for user-supplied sequences	Web	https://www.bioinformatics.org/sms2/codon_usage.html

Given the rate at which bacterial genomes are now being produced daily, a static resource has limited utility. Although CoCoPUTs integrates all complete genomes in GenBank, it does not include bacterial assemblies, the largest growing collection of bacterial genomic sequences. Furthermore, these databases must be updated by the tool’s team. The Dynamic Codon Biaser (DCB) was developed to facilitate statistical analysis of codon usage bias across different bacterial genomes using the 40 different highly conserved and highly expressed genes described by Sharp *et al*. [4]. This web application is dynamic and will identify HEG sequences and calculate results in real time for both publicly available genomes (draft or complete), by querying NCBI’s GenBank directly, as well as user-supplied genome sequences.

## Implementation

The DCB was developed in Python 3 utilizing completely open source tools and software libraries including Prodigal [36], DIAMOND [37], Biopython [38], Beautifulsoup4 (https://www.crummy.com/software/BeautifulSoup/) and Flask (http://flask.pocoo.org/). DIAMOND and Prodigal identify HEGs and annotate genome sequences, respectively. While there are many homology tools available, DIAMOND was selected for its speed. Python flask was chosen as the web framework as it requires minimal support and is lightweight and scalable. The DCB webserver is available at www.cbdb.info and source code is available at https://github.com/BrianDehlinger/DCB-Dynamic-Codon-Biaser.

**Fig. 1.**
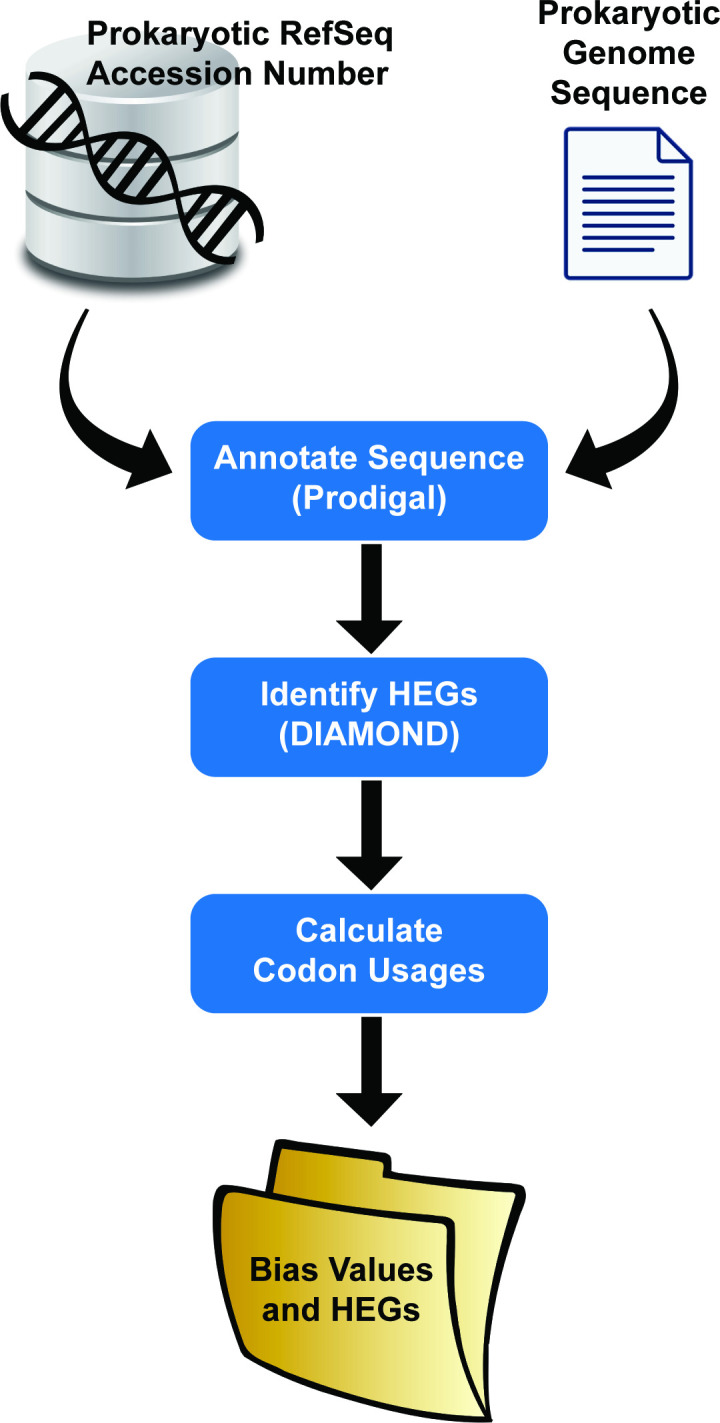
Basic workflows for DCB.

Fig. 1 outlines the process for analysis using either publicly available sequences from NCBI (left) or user-supplied genome sequences (right). For NCBI sequences, the web application accepts a RefSeq accession number as input. This can be either a complete genome or draft genome assembly. Beautifulsoup4 is used to navigate NCBI’s ftp back end and retrieve the organism’s assembly and annotated coding sequences from the genome (*.fna files). If the genome sequence is not annotated, Prodigal is run to identify coding regions. DIAMOND then queries coding sequences (from either NCBI’s annotation or Prodigal predictions) against a local protein database containing representatives of the 40 HEGs described by Sharp *et al*. [4]. This database was constructed by utilizing the Identical Protein Groups tool on NCBI and filtering to only include prokaryotes from the UniProtKB/Swiss-Prot source database [39]. The final database size included 1186 different sequences, representative of the phylogenetic diversity of sequenced prokaryotic species. This database can be retrieved via our GitHub repository, https://githubcom/BrianDehlinger/DCB-Dynamic-Codon-Biaser./blob/master/testApp/protein_databasefasta. The top DIAMOND hit for each of the 40 HEGs is identified and codon usage is calculated using a modified version of Biopython’s CodonUsage module [38]. DCB reports three statistics related to codon usage: the relative synonymous codon usage (RSCU), the normalized relative synonymous codon usage (NRSCU) and frequency bias (HEG FB). The web application returns a zip file containing the statistics in addition to the HEG file. Statistics are written in comma-separated value (csv) files to facilitate analysis via Excel or Python or R.

Computer code providing the same functionality available through the web is also available through our GitHub repository. Users can install and run the tool locally or set up their own web service. This code was developed using Python, HTML, CSS and Javascript for Linux Ubuntu 14.04 or higher. Dependencies include Prodigal and the following Python modules: Biopython, Beautifulsoup4, Pandas, Numpy, flask, requests and flask-bootstrap.

## Results

To evaluate the utility of DCB, three investigations were conducted. First, we evaluated the codon usage of *Escherichia coli* str. K-12 substr. MG1655 (accession NZ_CP032667.1). Entering this accession number into the web interface, the results are quickly generated and automatically downloaded in a zipped folder. These results include two files: (1) a comma-separated value file listing the codon usage statistics within the HEGs and (2) a fasta format file listing the HEG sequences used to compute the codon usage statistics. Note, these usage statistics could be used to evaluate all coding sequences within the genome; such codon usage tables are required for several of the tools listed in Table 1, such as CAIcal. This genome was selected as it is also one of the 200 genomes available in HEG-DB, which identifies codon usage biases in ribosomal proteins and predicts gene expression of all genes in the genome based upon their usage of these biases. Furthermore, this strain is an available option in CoCoPUTs. While DCB provides codon usage metrics based upon HEGs, CoCoPUTs provides raw counts of usage for the entire sequence. In this first proof-of-concept example, we selected a complete genome sequence. In the event that we wanted to evaluate a draft assembly or a user-supplied sequence, neither HEG-DB nor CoCoPUTs would be capable of conducting this analysis.

**Fig. 2.**
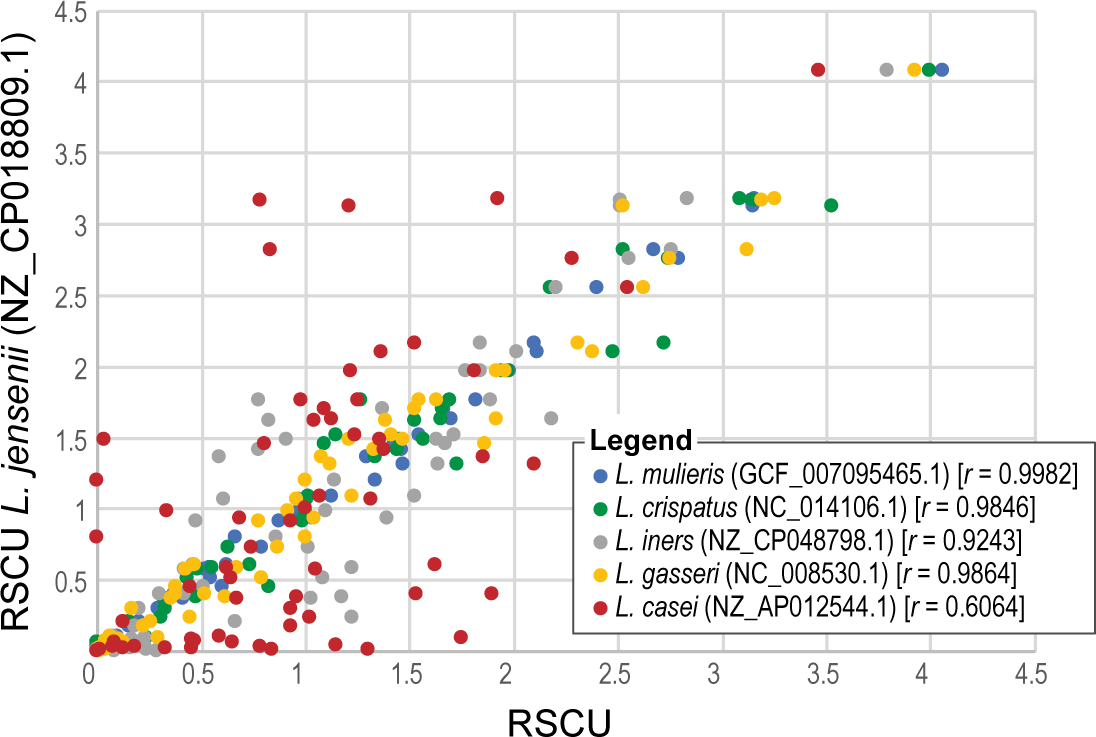
Comparison of codon usage biases in *Lactobacillus* species. Five genomes are compared to *L. jensenii* (NZ_CP018809.1): *L. mulieris* (GCF_007095465.1), *L. crispatus* (NC_014106.1), *L. iners* (NZ_CP048798.1), *L. gasseri* (NC_008530.1) and *L. casei* (NZ_AP012544.1).

In our second investigation, we focused on comparison of several draft and complete assemblies. In March 2020 a new species of lactobacilli was identified, *Lactobacillus mulieris* [40], which also resulted in taxonomic reclassification of several *Lactobacillus jensenii* strains [40, 41]. Using DCB, we wanted to examine the codon usage profiles of *L. mulieris* and *L. jensenii* as well as other *Lactobacillus* species of the urogenital tract and lactobacilli of the gut. As expected, *L. mulieris* and *L. jensenii* have the most similar codon usage of the species examined (Fig. 2). Furthermore, *L. jensenii* has a codon usage more similar to other members of the *Lactobacillus delbrueckii* group, which are also found in the urogenital microbiota, than the *Lactobacillus casei* group (represented by *L. casei* from the gut microbiota). Generally speaking, the observed variation in codon usage mirrors other phylogenetic markers for the genus [42]. An open question, however, is how does the different environments in which these strains are found (urogenital vs. gut) shape codon usage. Previous metagenomic studies have noted such adaptations of entire microbial communities to their environments [43].

In our third proof-of-concept example, we returned to our original motivation behind exploring codon usage in bacteria – phages. Phage Pbunalikevirus phiHabibi was isolated from Lake Michigan and found to be able to lyse both *Pseudomonas aeruginosa* ATCC 15692 and *E. coli* C [44]. Codon usage biases were calculated by DCB for these two hosts, accession numbers NC_010468 and NZ_CP017149, respectively. RSCU values for each host were compared to RSCU values calculated for the phage coding regions individually and collectively (accession number KT254132). Python code to perform this calculation is provided in File S1 (available in the online version of this article). phiHabibi had a codon usage bias more similar to the *P. aeruginosa* HEGs (*r*=0.8205) than the *E. coli* HEGs (*r*=0.5500), suggesting that *P. aeruginosa* is more likely to be the native or frequent host of this isolated phage. In total, 85 of the 90 protein coding genes similarly exhibited a codon usage more similar to *P. aeruginosa* HEGs (Table S1). Most notable is the similarity in codon usage between the *P. aeruginosa* HEGs and phiHabibi structural proteins. Previous bioinformatic analyses of phages and hosts have made similar observations [20]. Conducting such codon usage comparisons can also be useful for engineering phages; previous work has shown that codon optimization and deoptimization can increase and decrease, respectively, phage fitness [45, 46]. Given the recent renewed interest in phages for therapeutic use, codon usage is a promising avenue for phage engineering [47, 48].

DCB was specifically designed such that it has access to the latest publicly available complete and draft genomes; it is not dependent upon database updates, but rather it directly retrieves data from NCBI. Furthermore, it is flexible, allowing the user to upload a FASTA format file directly and does not require the user to supply HEGs; they are automatically detected for unannotated sequences, and HEG sequences are returned for all searches. For the calculation of a single genome, DCB uses around 70–75 MB of HDD space and around 72 MB of RAM. These run-time and memory usage statistics are informative for those users interested in running the tool locally rather than via the webserver, which is hosted as an EC2 instance. The source code is written to utilize one CPU core but allows for concurrent requests if a concurrent WSGI server is used as a wrapper for the flask server.

## Conclusion

DCB provides codon usage bias analysis for all publicly available or user-supplied prokaryotic genomes. The program is available as a web application with source code available for those users interested in running it locally. The program is also modular and can be modified and expanded upon to meet different use cases. Because calculations can be generated in a matter of seconds and new prokaryotic genomes are being deposited in GenBank daily, a dynamic web service provides a better solution than static databases. Data generated from DCB analyses can easily be integrated into, for example, evolutionary studies of prokaryotes, comparative genomic studies and phage–host investigations.

## Supplementary Data

Supplementary material 1
