# Supplementary material for: The Dynamic Codon Biaser: calculating prokaryotic codon usage biases
Source: Microb Genom. 2021 Oct 26;7(10):000663. doi: 10.1099/mgen.0.000663 (PMC8627211; doi:10.1099/mgen.0.000663)
Supplement: Supplementary material 1 [file mgen-7-0663-s001.pdf]

Supplemental Table 1: Correlation in codon usage between Pbunalikevirus phiHabibi coding regions and bacterial hosts

Similarity to E. coli HEG usage (r)	Similarity to P. aeruginosa HEG usage (r)	Protein Product	Protein_ID
0.503955282	0.580544806	hypothetical protein	ALJ99356.1
0.433206811	0.667079498	hypothetical protein	ALJ99357.1
0.128648028	0.396379896	hypothetical protein	ALJ99358.1
0.346952941	0.52179011	terminase large subunit	ALJ99359.1
0.457925069	0.631294184	hypothetical protein	ALJ99360.1
0.207783868	0.233809023	hypothetical protein	ALJ99361.1
0.375697709	0.558241746	hypothetical protein	ALJ99362.1
-0.106963675	0.171327868	hypothetical protein	ALJ99363.1
0.680349657	0.804728289	hypothetical protein	ALJ99364.1
0.269278416	0.514386646	hypothetical protein	ALJ99365.1
0.324034388	0.436662895	hypothetical protein	ALJ99366.1
0.523984132	0.730455113	hypothetical protein	ALJ99367.1
0.183437838	0.31058946	hypothetical protein	ALJ99368.1
0.139409283	0.222772586	hypothetical protein	ALJ99369.1
0.261967563	0.255799653	hypothetical protein	ALJ99370.1
0.23094794	0.256192602	hypothetical protein	ALJ99371.1
0.363454556	0.351072397	hypothetical protein	ALJ99372.1
0.4216468	0.625015876	putative minor head protein	ALJ99373.1
0.05013603	0.194823509	minor capsid protein	ALJ99374.1
0.14205873	0.182175143	hypothetical protein	ALJ99375.1
0.147344164	0.353984387	hypothetical protein	ALJ99376.1
0.726350304	0.858591988	putative structural protein	ALJ99377.1
0.645158955	0.836559438	putative structural protein	ALJ99378.1
0.646718428	0.904364602	capsid and scaffold protein	ALJ99379.1
0.585207818	0.673119851	hypothetical protein	ALJ99380.1
0.301943299	0.359939177	putative structural protein	ALJ99381.1
0.205282252	0.198325382	putative structural protein	ALJ99382.1
0.127764357	0.260818002	hypothetical protein	ALJ99383.1
0.143132943	0.078913882	hypothetical protein	ALJ99384.1
0.585886304	0.848845823	putative structural protein	ALJ99385.1
0.440546646	0.570055867	hypothetical protein	ALJ99386.1

Supplemental Table 1: Correlation in codon usage between Pbunalikevirus phiHabibi coding regions and bacterial hosts

Similarity to E. coli HEG usage (r)	Similarity to P. aeruginosa HEG usage (r)	Protein Product	Protein_ID
0.321817839	0.455024819	putative structural protein	ALJ99387.1
-0.024467178	0.203021829	putative structural protein	ALJ99388.1
0.461642621	0.656693169	hypothetical protein	ALJ99389.1
0.177836992	0.284959979	putative structural protein	ALJ99390.1
0.169735028	0.273862718	putative structural protein	ALJ99391.1
-0.010292109	0.119120729	tail fiber protein	ALJ99392.1
0.332667308	0.517468142	hypothetical protein	ALJ99393.1
0.275320123	0.461872565	internal (core) protein	ALJ99394.1
0.368543259	0.495159712	putative structural protein	ALJ99395.1
-0.062129033	-0.02842687	hypothetical protein	ALJ99396.1
0.365967195	0.499868144	putative baseplate protein	ALJ99397.1
0.406545459	0.587692862	hypothetical protein	ALJ99398.1
0.569139917	0.705775094	putative structural protein	ALJ99399.1
0.539922564	0.731780067	tail fiber protein	ALJ99400.1
0.210875851	0.337204168	tail fiber component	ALJ99401.1
0.471872767	0.704060748	endolysin	ALJ99402.1
0.133404355	0.19315196	hypothetical protein	ALJ99403.1
0.394846674	0.654027288	DNA ligase	ALJ99404.1
0.396161404	0.707553728	DNA-binding protein	ALJ99405.1
0.346331695	0.506540289	hypothetical protein	ALJ99406.1
0.683100817	0.882543718	hypothetical protein	ALJ99407.1
0.504068507	0.700678089	hypothetical protein	ALJ99408.1
0.561994006	0.673366097	DNA helicase	ALJ99409.1
0.246955068	0.457251128	hypothetical protein	ALJ99410.1
0.254160635	0.502374104	DNA polymerase III alpha subunit	ALJ99411.1
0.098542316	0.260979512	DNA polymerase III epsilon subunit	ALJ99412.1
0.514077269	0.777039972	3'-phosphatase, 5'-polynucleotide kinase	ALJ99413.1
0.488060284	0.612556862	hypothetical protein	ALJ99414.1
0.442805147	0.599262117	thymidylate synthase	ALJ99415.1
0.307815263	0.362867802	hypothetical protein	ALJ99416.1
0.366835833	0.448256198	hypothetical protein	ALJ99417.1

Supplemental Table 1: Correlation in codon usage between Pbunalikevirus phiHabibi coding regions and bacterial hosts

Similarity to E. coli HEG usage (r)	Similarity to P. aeruginosa HEG usage (r)	Protein Product	Protein_ID
0.347878792	0.355106045	tail assembly protein	ALJ99418.1
0.173475073	0.328298584	hypothetical protein	ALJ99419.1
0.231716238	0.486664936	hypothetical protein	ALJ99420.1
0.509303376	0.75313338	hypothetical protein	ALJ99421.1
0.65437547	0.893267682	putative structural protein	ALJ99422.1
0.255728406	0.398960031	DNA helicase	ALJ99423.1
0.319131732	0.350491653	hypothetical protein	ALJ99424.1
0.379478032	0.595046982	hypothetical protein	ALJ99425.1
0.5012435	0.612454833	hypothetical protein	ALJ99426.1
0.241132704	0.447812447	hypothetical protein	ALJ99427.1
0.089109196	0.440124754	hypothetical protein	ALJ99428.1
0.127048177	0.130950242	hypothetical protein	ALJ99429.1
0.275706177	0.499520718	DNA primase	ALJ99430.1
0.561562512	0.775240847	hypothetical protein	ALJ99431.1
0.676684212	0.864472133	hypothetical protein	ALJ99432.1
0.176486402	0.287260014	hypothetical protein	ALJ99433.1
0.230921919	0.41073471	hypothetical protein	ALJ99434.1
0.544737363	0.426115926	hypothetical protein	ALJ99435.1
0.44739334	0.629965381	hypothetical protein	ALJ99436.1
0.364487115	0.397045876	hypothetical protein	ALJ99437.1
0.401680545	0.623085987	hypothetical protein	ALJ99438.1
0.33570932	0.549102371	hypothetical protein	ALJ99439.1
0.1408467	0.233054455	hypothetical protein	ALJ99440.1
0.383463927	0.629648787	hypothetical protein	ALJ99441.1
0.310402628	0.444091189	hypothetical protein	ALJ99442.1
0.238489051	0.333467244	hypothetical protein	ALJ99443.1
0.184111171	0.288456919	hypothetical protein	ALJ99444.1
0.084609628	0.334095798	tail length tape-measure protein	ALJ99445.1

Supplemental File 1. Python script to compute phage to bacterial host RSCU comparisons

```
#!/usr/bin/env python3
# -*- coding: utf-8 -*-

#
================================================================
====
# This code takes in a multi-FASTA format file for phage coding regions
and
# a bacterial codon usage file from cbdb.info (DCB).
#
# To execute,
# python calc_phage_DCB.py phage_file bacterial_file output_file
#
# phage_file: multi-FASTA format file for coding sequences
# bacterial_file: DCB format output file from cbdb.info
# output_file: name of file for results of calculation
#
================================================================
====


from Bio import SeqIO
import sys

# retrieve parameters from the command line
if len(sys.argv)!=4:
    sys.exit('ERROR: To execute code, provide 3 arguements:\n  - path of
phage coding sequences FASTA nucleotide file\n  - bacterial codon usage
file from CBDB\n  - output file name\n')
phage_file=sys.argv[1]
bacterial_file=sys.argv[2]
out_file=sys.argv[3]

# calculate rscu
def rscu(x):
    aa_table = {
            'ATA':'I', 'ATC':'I', 'ATT':'I', 'ATG':'M',
            'ACA':'T', 'ACC':'T', 'ACG':'T', 'ACT':'T',
            'AAC':'N', 'AAT':'N', 'AAA':'K', 'AAG':'K',
            'AGC':'S', 'AGT':'S', 'AGA':'R', 'AGG':'R',
            'CTA':'L', 'CTC':'L', 'CTG':'L', 'CTT':'L',
            'CCA':'P', 'CCC':'P', 'CCG':'P', 'CCT':'P',
            'CAC':'H', 'CAT':'H', 'CAA':'Q', 'CAG':'Q',
            'CGA':'R', 'CGC':'R', 'CGG':'R', 'CGT':'R',
            'GTA':'V', 'GTC':'V', 'GTG':'V', 'GTT':'V',
            'GCA':'A', 'GCC':'A', 'GCG':'A', 'GCT':'A',
            'GAC':'D', 'GAT':'D', 'GAA':'E', 'GAG':'E',
            'GGA':'G', 'GGC':'G', 'GGG':'G', 'GGT':'G',
            'TCA':'S', 'TCC':'S', 'TCG':'S', 'TCT':'S',
            'TTC':'F', 'TTT':'F', 'TTA':'L', 'TTG':'L',
            'TAC':'Y', 'TAT':'Y', 'TAA':'_', 'TAG':'_',
```

```
            'TGC':'C', 'TGT':'C', 'TGA':'_', 'TGG':'W',
        }

    aas=set(aa_table.values())
    aa_count={i: 0 for i in aas}
    aa_codon_counts={i: 0 for i in aas}
    for i in aa_table:
        aa_codon_counts[aa_table[i]]+=1

    for i in x:
        aa_count[aa_table[i]]+=x[i]

    y={}
    for i in x:
        try:

y[i]=str(float(x[i])/float(aa_count[aa_table[i]])*aa_codon_counts[aa_tabl
e[i]])
        except:
            y[i]='0'
    return y

# phage genome sequence
genes=list(SeqIO.parse(phage_file,'fasta'))

# bacterial genome sequence
with open(bacterial_file,'r') as f:
    lines=f.readlines()

# store bacterial values from CBDB
codons=[]
bacteria={}
for i in lines[1:]:
    l=i.split(',')
    codons.append(l[0])
    bacteria[l[0]]=float(l[1])

# get codon counts from phage nucleotide FASTA file for each coding
region
g=[]
for i in genes:
    c={i: 0 for i in codons}
    for j in range(0,len(str(i.seq)),3):
        x=str(i.seq)[j:j+3]
        try:
            c[x]+=1
        except:
            continue
    g.append(c)

# calculate usage for all coding regions
all_phage={i: 0 for i in codons}
for i in g:
    for j in all_phage:
```

```
        all_phage[j]+=i[j]
rscu_all_phage=rscu(all_phage)

# write out results
outfile=open(out_file,'w')
e=rscu(bacteria)
outfile.write('All Coding
Regions'+','+','.join(rscu_all_phage.keys())+'\n')
outfile.write(bacterial_file+','+','.join(e.values())+'\n')
outfile.write(phage_file+','+','.join(rscu_all_phage.values())+'\n\n')

outfile.write('Individual Coding
Regions'+','+','.join(rscu_all_phage.keys())+'\n')
for i in range(len(g)):
    x=rscu(g[i])

outfile.write(str(genes[i].description)+','+','.join(x.values())+'\n')

outfile.close()
```
